# Multidataset Refinement Resonant Diffraction, and Magnetic Structures

**DOI:** 10.6028/jres.109.007

**Published:** 2004-02-01

**Authors:** J. Paul Attfield

**Affiliations:** Department of Chemistry, University of Cambridge, Lensfield Road, Cambridge CB2, 1EW and Interdisciplinary Research Centre in Superconductivity, Department of Physics, University of Cambridge, Madingley Road, Cambridge CB3 0HE.

**Keywords:** crystal structure, magnetic structure, multidataset refinement, multiphase analysis, powder neutron diffraction, powder x-ray diffraction, Rietveld refinement

## Abstract

The scope of Rietveld and other powder diffraction refinements continues to expand, driven by improvements in instrumentation, methodology and software. This will be illustrated by examples from our research in recent years. Multidataset refinement is now commonplace; the datasets may be from different detectors, e.g., in a time-of-flight experiment, or from separate experiments, such as at several x-ray energies giving resonant information. The complementary use of x rays and neutrons is exemplified by a recent combined refinement of the monoclinic superstructure of magnetite, Fe_3_O_4_, below the 122 K Verwey transition, which reveals evidence for Fe^2+^/Fe^3+^ charge ordering. Powder neutron diffraction data continue to be used for the solution and Rietveld refinement of magnetic structures. Time-of-flight instruments on cold neutron sources can produce data that have a high intensity and good resolution at high *d*-spacings. Such profiles have been used to study incommensurate magnetic structures such as FeAsO_4_ and β–CrPO_4_. A multiphase, multidataset refinement of the phase-separated perovskite (Pr_0.35_Y_0.07_Th_0.04_Ca_0.04_Sr_0.5_)MnO_3_ has been used to fit three components with different crystal and magnetic structures at low temperatures.

## 1. Introduction

This paper describes some of the useful extensions of the Rietveld profile analysis method [1]. The Rietveld method has been widely used to analyse powder diffraction data for more than 30 years. In essence, the method is a fit to a series of points that describe measured diffraction intensity as a function of 2*θ*, neutron time-of-flight, or other measures of scattering vector. The calculated function describes the scattered background and the position, shape and intensities of the Bragg diffraction peaks. Optimization of the fit between calculated and observed intensities is usually achieved by least-squares refinement of parameters in the calculated function. Some of these are fundamental to the crystal structure and other parameters, particularly in the peak shape, describe the sample microstructure (through strain or particle-size broadening) and the diffractometer resolution function.

Like all fitting procedures, a Rietveld fit can be biased by systematic differences between the observed and calculated data arising from poor data, e.g., from granular samples, or from deficiencies in the model, e.g., inadequate background or peak shape functions, unmodelled secondary phases, incorrect space groups or misplaced atoms, leading to results of uncertain accuracy. However, in very many cases the systematic errors are small, and statistically good (and visually impressive) fits to data sets containing many hundreds or thousands of points are obtained. The freely refined parameters can be both precise and accurate. In such cases, the limitations of the Rietveld refinement arise from the sample complexity and quality, and from the diffraction experiment. When the sample quality is good and the best available diffractometer has been used, then further information about the sample may still be gained by introducing more observations. These may be in the form of a second diffraction dataset containing significant information that is not present in the original pattern, or may be another type of information, such as geometric restraints. Examples of these types of refinement are presented below. The following section describes some refinements of magnetic structures, many of which now benefit from the inclusion of multiple datasets. The examples are taken from recent studies within the author's research group. Many similar refinements are reported in the literature, this paper is not intended as a review of all work in the area.

## 2. Multidataset Refinements

### 2.1 Multiple Detectors

Powder diffraction intensity is often recorded simultaneously by several detectors in order to improve counting statistics. In many angle-dispersive experiments that use a moving bank of equivalent detectors, the individual datasets from each detector can be summed together, with some corrections for different counting efficiencies, to give a single dataset for subsequent Rietveld analysis. However, this approach is not appropriate for time-of-flight neutron diffraction for which the measured time of flight *t* is related to *d*-spacing *d* as;

t=(2mL/h)dsinθ

where

$$\begin{array}{l} m=\text{neutron mass}\\ L=\text{neutron flight path}\\ h=\text{Planck's constant}\\ \theta =\text{scattering angle} \end{array}$$

and the time resolution *dt/t* is given by:

dt/t=(dL/L)+(cotθ)dθ.

Time-of-flight experiments are usually performed at pulsed neutron sources so that the range of observed times is limited by the pulse width and repetition rate. This limits the range of observed *d*-spacings. To extend this range, a dataset from a detector observing the sample over the same time interval but at a different *θ* angle may be used, but this has a different resolution because of the cot *θ* term in the resolution function, so that the two data sets cannot easily be summed together. This led to the first popular use of multidataset refinements through the GSAS software [2]. Each dataset is fitted with an individual background and peak shape function, but the sample parameters are common to all the fits. Refinements using patterns from several detectors are common (in practise, each “detector” is itself a bank of several elements over a narrow angular spread that are time-focused to behave as a single detector). As an example, the recently commissioned GEM diffractometer [3] at the ISIS pulsed neutron facility in the UK will have eight detector banks between 1° and 169° 2*θ*, containing a total of 8000 individual detector elements.

### 2.2 Multiple Experiments—Elemental Contrast

Software for Rietveld analysis of multiple datasets can also be applied to data from different experiments on the same sample. The most common use of these experiments is to exploit elemental contrast between different radiations, typically x ray/neutron or x ray/x ray in which resonant contrast is obtained at different wavelengths. Neutron/neutron contrast can be obtained through isotopic substitution, requiring two samples prepared under the same conditions. Such refinements are used to give accurate atomic parameters (coordinates, temperature factors, site occupancies) for specific elements, particularly in disordered materials.

#### 2.2.1 X-Ray/Neutron Contrast

The differences between the atomic number variations of x-ray and neutron scattering factors of the elements give good contrast in many cases. A past example is a structural study of the supposed ternary oxide “BaCuO_2_” [4]. This has a large cubic cell (space group I*m*3*m*, *a* = 18.307 Å) with disorder of many of the copper and oxygen sites. Analysis using x-ray and neutron data sets together showed that a supposed metal cation site was occupied by carbon, and that the material is an oxycarbonate Ba_44_Cu_48_(CO_3_)_6_O_88_. The ratios of x-ray to neutron (*b* in fm) scattering factors for Ba, Cu and C are 10.7, 3.8, and 1.4, respectively, enabling the carbon atom to be assigned with confidence. Although the two datasets (from a commercial laboratory x-ray diffractometer and a medium flux reactor instrument) were of moderate quality, their combination gave important chemical and structural information that had been missed in previous single crystal and powder diffraction studies using individual datasets of much higher quality.

A recent example that makes use of state-of-the-art x-ray and neutron powder diffraction data is a refinement of magnetite below the Verwey transition [5]. This is an old and difficult structural problem. At ambient temperatures, Fe_3_O_4_ has the cubic, inverse spinel crystal structure (space group *m*, *a* = 8.39 Å). This has Fe^III^ on the (A type) tetrahedral cation sites whereas the Fe^II^ and Fe^III^ occupy the two (B type) octahedral sites. The incomplete cancellation of the two B site magnetic moments by the antiparallel A site cation moment results in ferrimagnetism, and electron delocalisation or hopping between the B site Fe^II^ and Fe^III^ ions renders the B sites structurally and spectroscopically equivalent, and gives rise to a moderate electronic conductivity. In 1939, Verwey discovered that magnetite undergoes a sharp, first order transition on cooling below 120 K, at which the resistivity of magnetite increases sharply by two orders of magnitude, and the structure distorts from cubic symmetry. This is consistent with charge ordering of the Fe^II^ and Fe^III^ states on the B sublattice and an orthorhombic superstructure model was proposed [6]. However, this was not confirmed by subsequent single crystal studies, which were hampered by the severe twinning that accompanies the transition to the low temperature structure which has a √2*a* × √2*a* × 2*a* superstructure with monoclinic *Cc* (or lower) symmetry. The most detailed neutron structure refinement used a magnetically aligned, mechanically detwinned, single crystal [7]. An *a*/√2 × *a*/√2 × 2*a* subcell of the above cell with orthorhombic P*mca* or P*mc*2_1_ symmetry constraints was used to reduce the number of variables in the refined models. No charge ordered arrangement was identified in the refined structures.

Powder diffraction experiments avoid the twinning and multiple scattering problems that have beset studies of magnetite crystals below the Verwey transition. However, the higher background and peak overlap inherent to powder experiments are the compensating disadvantages. Both high resolution and a high peak-to-background intensity ratio are needed, as the metric distortion is very small (equivalent to ≈0.2° in the 90° angle of the cubic F*d*3*m* cell) and the superstructure peaks have <1 % of the intensities of the fundamental reflections. The refinement made use of a highly stoichiometric sample of magnetite (kindly provided by Prof. J. Honig, Purdue University) and very high-resolution neutron and x-ray powder diffractometers. Data were collected at 130 K and 90 K, the Verwey transition occurs at 122 K in this sample. Neutron data in the range *d* = 0.31 Å to 4.45 Å were obtained from the backscattering detector bank on HRPD at the ISIS spallation source, UK. Synchrotron x-ray powder data from a spinning 0.7 mm capillary were collected using the BM16 instrument^1^ at ESRF with a wavelength of 0.49395 Å up to 2*θ* = 70° (*d* = 0.43 Å). The GSAS package [2] was used for Rietveld fits to the data.

**Fig. 1:**
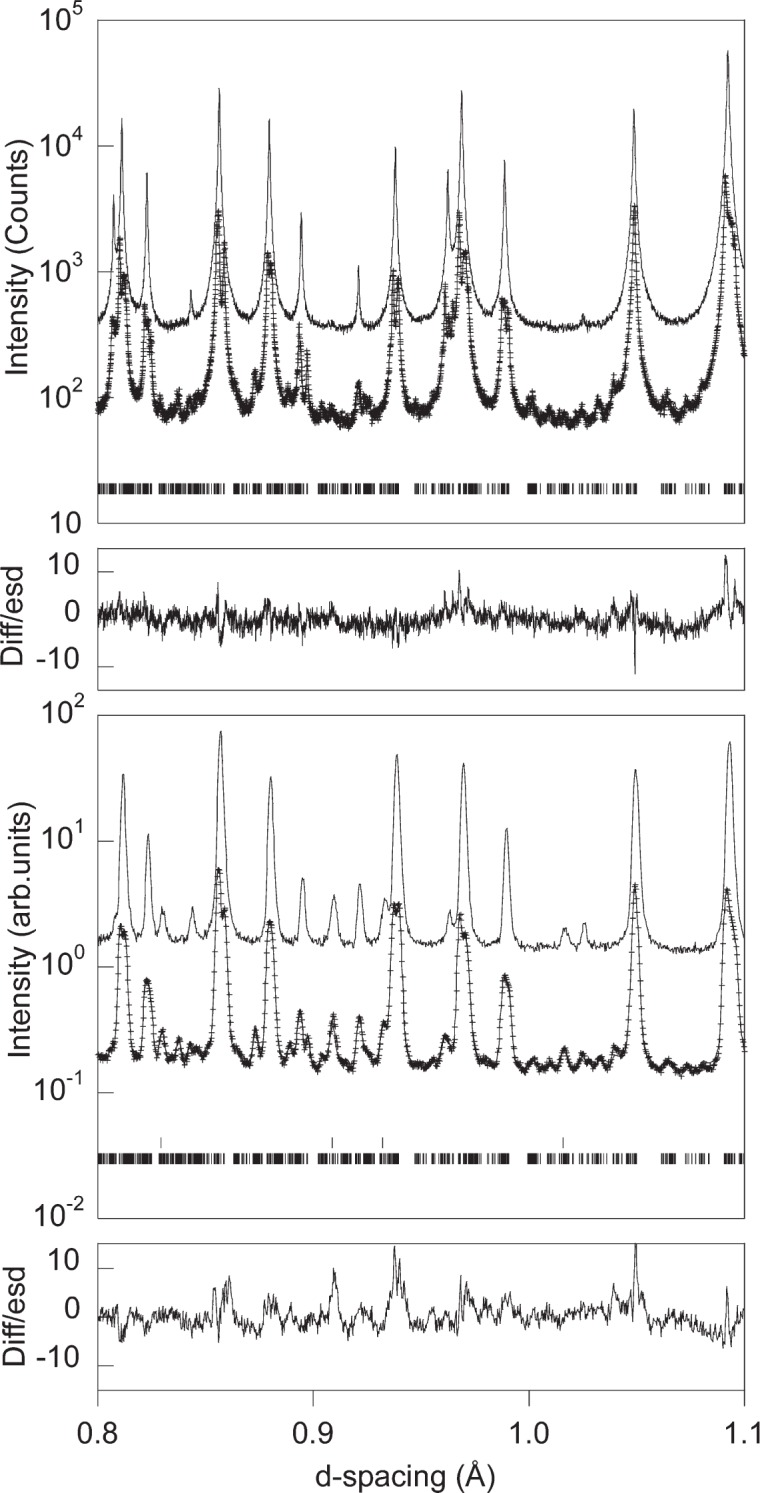
Part of the fitted powder x-ray (upper panels) and neutron (lower panels) diffraction patterns from the multipattern refinement of Fe_3_O_4_. The intensity scales are logarithmic in order to emphasise the weak superstructure peaks. Observed (crosses), calculated (full lines) and difference (as difference/estimated standard deviation) plots are shown for the fit to the 90 K data. The observed patterns at 130 K (above the Verwey transition) are also plotted one decade above the 90 K data. Markers show the positions of the Bragg reflections in the low temperature Fe_3_O_4_ structure, markers for aluminium in the sample environment are also shown for the neutron data.

Separate fits of monoclinic models with the *a*/√2 × *a*/√2 × 2*a* subcell to the 90 K x-ray and neutron profiles were not stable so orthorhombic symmetry constraints were applied. It was found that the *Pmca* symmetry constraints used previously by Iizumi et al. [7] gave the best fits and convergent refinements. The refinements in *Pmca* were found to be robust and returned to the same minimum after atoms were displaced away from the refined positions. In the final stages, a combined x-ray and neutron refinement was carried out. The difference between the ratios of neutron scattering factors (*b*_Fe_/*b*_O_ = 1.6) and x-ray form factors (*f*_Fe_/*f*_O_ ≈ 3) reduces correlations between the refined parameters. The values and errors of the atomic coordinates in the final *Pmca*- constrained model are similar to those from the previous single crystal study. Part of the fitted profiles is shown in Fig. 1. Full results of the refinement will be given elsewhere [8].

The refinement of the low temperature structure of magnetite from powder data is challenging because of the symmetry is lowered greatly at the Verwey transition. The cubic spinel structure has 3 unique atoms and 1 variable coordinate, while the *Pmca*-constrained subcell has 16 atoms and 23 variable coordinates (and the “true” *Cc* structure has an asymmetric unit that is four times larger). Despite this complexity, our study has shown that state-of-the-art powder x-ray and neutron data can give refinements that are accurate (insofar as the refined parameters from the individual refinements and the previous neutron study are consistent) and precise (in that the combined refinement gives errors that are comparable with the single crystal study).

Although the refined coordinates are similar to those from the previous single crystal study [7], some differences between the Fe–O distances from the two refinements are apparent. The *Pmca*-constrained model contains four independent octahedral B sites, two of which have significantly longer Fe–O distances [2.069(4) and 2.072(3) Å] than the other two [(2.043(3) and 2.050(4) Å)] in the powder refinement. This has provided direct crystallographic evidence for charge order below the Verwey transition, although the magnitude of the estimated charge disproportionation is only 20 % of that expected for ideal Fe^2+^ and Fe^3+^ states.

#### 2.2.2 X-Ray/X-Ray Contrast

Elemental contrast can be exploited in multidataset Rietveld refinements through resonant x-ray scattering (anomalous dispersion). This topic was previously reviewed in detail [9]. Large real (*f*′) and imaginary (*f*″) contributions to the x-ray scattering factor arise at energies close to an elemental absorption edge. A useful strategy in powder diffraction studies is to tune the x-ray energy to 5 eV to 20 eV below the edge. This gives large, negative values of *f*′, but with small sample absorption and *f*″ corrections. This strategy has been used to identify and quantify cation disorder in many complex solids, for example, the thallium cuprate superconductors Tl_2_Ba_2_CuO_6_ [10] and Tl_0.5_Pb_0.5_Sr_2_Ca_2_Cu_3_O_9_ [11]. In the latter example, resonant datasets at the Tl L_III_, Pb L_III_, Sr K and Cu K edges were combined with neutron data (to give accurate oxygen positions) in the simultaneous refinement. The final refinement enabled the site by site composition to be given as (Tl_0.60(2)_Pb_0.40_)(Sr_1.60(2)_Ca_1.40_)(Ca_1.93(1)_Tl_0.07_)Cu_3_O_9_, earlier refinements having ruled out other possible substitutions such as Cu at the Tl site or Pb at the Ca site.

Previous resonant powder diffraction studies also showed that different electronic states of the same element can be distinguished on the basis of their anomalous scattering, e.g., Eu^2+^ and Eu^3+^ in Eu_3_O_4_ [12]. This has become known as the DAFS (differential anomalous fine structure) technique which is essentially site-resolved XAFS spectroscopy. Although it was demonstrated that this is feasible with powders [13], most DAFS studies use single crystals, e.g., in a recent study of Fe_3_O_4_ [14]. For a recent review of resonant diffraction studies see [15].

#### 2.2.3 Neutron/Neutron Contrast

Several isotopes have large resonant neutron scattering terms at thermal energies and can, in principle, be used to achieve elemental contrast as a function of the neutron energy. However, this is very rarely used, as the wavelength variation is more gradual than for x-ray resonant scattering, and the absorption is more severe. The differences in scattering factors between different isotopes of the same element are more useful and can be used for elemental contrast experiments.

Isotopic contrast is particularly useful for light elements (*Z* < 20) for which resonant x-ray scattering is inapplicable, as the absorption edges are too low in energy for practical diffraction experiments. A single neutron diffraction pattern is often sufficient, but the need for multiple datasets is illustrated by the scattering properties of boron and carbon, which form many crystalline borocarbides. The neutron scattering lengths (*b*) and absorption cross sections (*σ*_abs_) for C and the principal B isotopes are *b*(C) = 6.65 fm, *σ*_abs_ (C) ≈ 0; *b*(^10^B) ≈ 0.4 fm, *σ*_abs_ (^10^B) = 755 × 10^−28^ m^2^; *b*(^11^B) = 6.66 fm, *σ*_abs_ (^11^B) = 0. Hence, C/^10^B samples give excellent elemental contrast but are strongly absorbing, whereas C/^11^B combinations are non-absorbing but give no contrast information. A multiprofile refinement using neutron patterns from both combinations can give precise atomic coordinates and distinguish between B and C positions. This approach was used to determine the B/C order within the tetragonal MB_2_C_2_ structure [16], which is adopted for M = Ca, lanthanides. The borocarbide sheets in this structure consist of fused 4 and 8-membered rings and two schemes for B/C order are apparent. The arrangement containing adjacent B_2_ and C_2_ pairs had been proposed from x-ray single crystal studies, although band structure calculations indicated that the alternative arrangement of alternating B and C atoms was more stable. The issue was settled by a combined neutron refinement using constant wavelength reactor data from both natural-B and ^11^B samples of CeB_2_C_2_. This clearly showed that the alternating B/C arrangement is correct.

### 2.3 Related Profiles

In the multidataset refinements described in Sec. 2.2, it is implicit that the sample is being observed under the same external conditions so that the intrinsic crystallographic parameters can be constrained to be equal across all the profile fits. An plausible extension of this method is to fit profiles collected under non-identical, but systematically varying conditions, such as changing temperature, pressure, time etc. This remains an underdeveloped aspect of Rietveld refinement. Automated sequential Rietveld analysis of related profiles data is commonly performed by using each refined model as the starting point for fitting the next dataset. The variation of refined parameters with external variables is then determined post-refinement. An example is the variable temperature study of manganese oxide perovskites AMnO_3_ [17] in which structural phase transitions and changes of average and local structure with temperature have been determined. Further developments in this area will require software to fit all of the datasets simultaneously, with user-input functions for the dependence of the refined parameters upon the external variable.

### 2.4 Other Observations

Multidataset analyses, in which the structure is fitted to both powder diffraction profile and other types of experimental observation, can be envisaged. In practice, the only type of “observed” data that are commonly fitted in addition to the profile intensities are geometric restraints such as ideal values of bond distances and angles, torsion angles, etc. It has recently been demonstrated that real space methods, e.g., the DASH software [18], for solving molecular crystal structures by global optimisation of profile fits can be highly successful. Geometrically restrained Rietveld refinements are then used to provide the best final model, although the number of restraints is usually small in comparison to the number of profile observations. An example is the structure determination of the pharmaceutical molecule tetracaine (C_15_N_2_O_2_H_25_^+^Cl^−^) [19]. Even protein structures such as the 1261 atom metmyoglobin have been refined from high-resolution synchrotron x-ray powder data [20]. In this case, the number of restraints (5338) was greater than the number of profile points (4648). In the future, it may be possible to include other types of experimental observation that contain structural information such as NMR spectra into multidataset refinements.

## 3. Magnetic Structures

The refinement of ordered magnetic structures using constant wavelength, angle-dispersive neutron diffraction data is well-established. In recent years it has become clear that time-of-flight data can give refinements of comparable quality. Good resolution and high flux at long *d*-spacings can be achieved on diffractometers with back-scattering geometry using cold neutron pulses, such as the IRIS and OSIRIS instruments at the ISIS spallation source. Low angle detectors on instruments utilising thermal neutron pulses can also be used, although the ∆*d*/*d* resolution is generally lower. Time-of-flight data have been used to solve the incommensurate magnetic structure of monoclinic FeAsO_4_ [21], and to obtain precise refinements of the spiral periodicity in β-CrPO_4_ and vanadate-doped samples [22]. The latter study showed that the spiral magnetic structure of β-CrPO_4_ is not triply commensurate with the lattice periodicity through a precise determination of the (*q_x_*,0,0) propagation vector. The refined *q_x_* = 0.3306(2) differs significantly from the commensurate *q_x_* = ⅓ value.

**Fig. 2:**
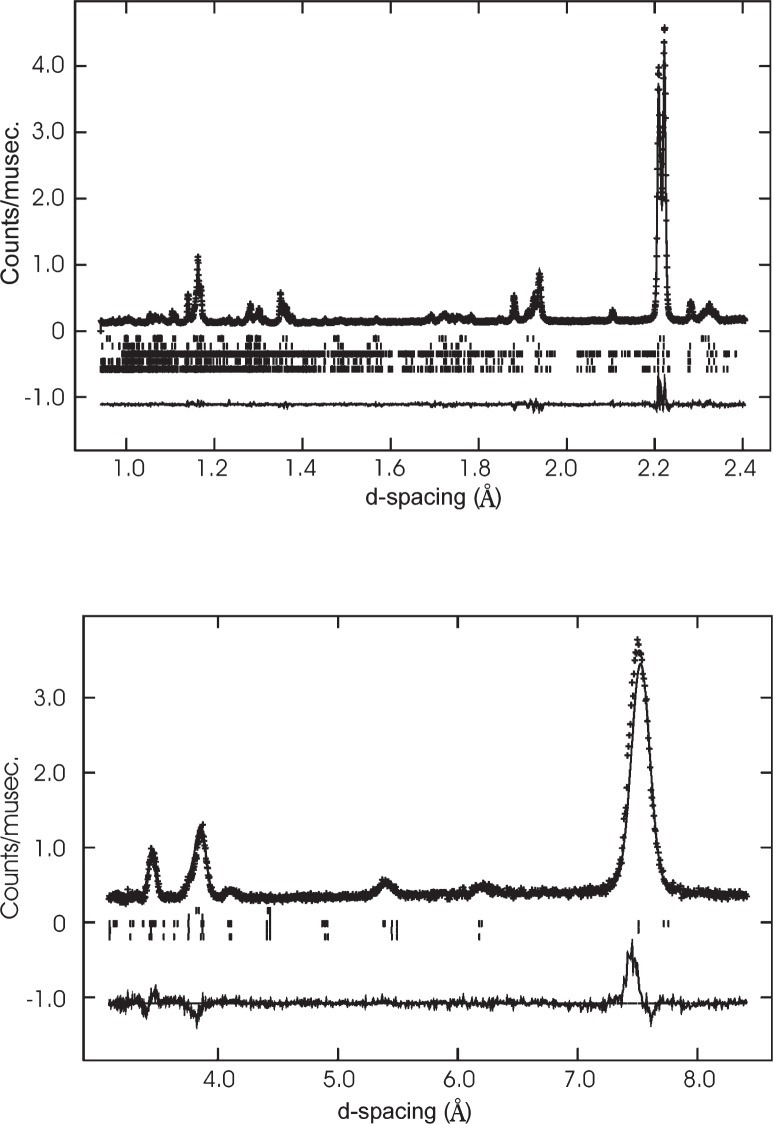
Observed, calculated and difference profiles for the datasets used for the three phase refinement of (Pr_0.35_Y_0.07_Th_0.04_Ca_0.04_Sr_0.5_)MnO_3_ at 2 K. The reflection markers from top to bottom are: F-type, A-type (nuclear), CE-type (magnetic), CE-type (nuclear), and A-type (magnetic).

Multipattern refinements can be used to combine backscattering data (to determine atomic coordinates precisely) with low angle bank data containing the magnetic peaks. A notable example is a recent refinement of (Pr_0.35_Y_0.07_Th_0.04_Ca_0.04_Sr_0.5_)MnO_3_, presented below. This 50 % doped manganite perovskite is a single phase at 300 K but separates into three different, magnetically ordered phases at low temperatures. One is ferromagnetic (F-type magnetic structure), the other two are both antiferromagnetic but one is charge ordered (CE-type), with distinct Mn^3+^ and Mn^4+^ sites, and the other is charge disordered (A-type). A refinement using 2 K data from the HRPD diffractometer at ISIS enabled the crystal structures of all three phases to be refined separately despite their similar cell metrics. Their very different magnetic diffraction peak distributions effectively fix the cell parameters for the three phases, enabling their nuclear contributions in the more heavily-overlapped low-*d* regions to be separated (Fig. 2). An indication that the results are accurate comes from the Mn-O bond distances which are in agreement with the distortions of the MnO_6_ octahedra expected from the orbital order associated with Jahn-Teller active Mn^3+^ (Table 1).

## 4. Conclusions

Continuing improvements in the speed and availability of x-ray (especially synchrotron) and neutron diffractometers, and computer hardware and software, have made multidataset refinements commonplace and have led to structural problems of increasing complexity being tackled by powder diffraction. The resulting parameters can have high precision and accuracy. Combined refinements with diffraction profile and other types of data are still rare, with the exception of restrained refinements which have enabled large molecules to be refined against powder data.

## Figures and Tables

**Table 1:** Observed Mn–O distances at 2 K compared against the expected distortions for each of the three low temperature magnetic perovskite phases in (Pr_0.35_Y_0.07_Th_0.04_Ca_0.04_Sr_0.5_)MnO_3_

Phase (and properties)	Expected MnO_6_ octahedral geometry	Observed Mn–O distances (Å)
F type (ferromagnetic, metallic)	Regular	1.936(1) × 4
		1.936(1) × 2
A type (antiferromagnetic, 2-dimensional conductor)	Tetragonal compression	1.901(1) × 2
		1.948(1) × 4
CE type (charge ordered, antiferromagnetic insulator)	Mn^3+^ site^a^: tetragonal elongation	1.900(3) × 2
		1.925(5) × 2
		2.056(6) × 2
	Mn^4+^ site^a^: regular	1.907(1) × 2
		1.916(1) × 2
		1.919(1) × 2

a The refinement was constrained to preserve the centres of symmetry of these octahedra.

## Footnotes

1 Certain commercial equipment, instruments, or materials are identified in this paper to foster understanding. Such identification does not imply recommmendation or endorsement by the National Institute of Standards and Technology, nor does it imply that the materials or equipment identified are necessarily the best available for the purpose.
